# Hepatosplenic Sarcoidosis Complicated by Liver Cirrhosis

**DOI:** 10.5334/jbr-btr.971

**Published:** 2015-12-30

**Authors:** B. Peters, F. M. Vanhoenacker, P. Bernard, H. Van Dijck, L. Van Overbeke

**Affiliations:** 1 Department of Radiology, AZ Sint-Maarten, Duffel-Mechelen; Rooienberg 25, B-2570 Duffel, Belgium; 2 Department of Radiology, Antwerp University Hospital, University of Antwerp, Edegem, Belgium; 3 Faculty of Medicine and Health Sciences, University of Ghent, Belgium

**Keywords:** Sarcoidosis, Spleen, Liver, MRI, CT

## Abstract

Sarcoidosis is a multisystemic disease usually affecting the lungs and mediastinal lymph nodes. Other organs, such as the liver and the spleen, are less commonly involved. Patients usually present with mild nonspecific symptoms. On imaging, hepatosplenomegaly with or without multiple focal lesions within the spleen may be seen in the active disease stage. Rarely, the disease may evolve to cirrhosis and liver failure. We report such a rare case of hepatosplenic sarcoidosis complicated by acute esophageal bleeding due to portal hypertension.

## Introduction

Sarcoidosis is a multisystemic disease usually affecting the lungs and mediastinal lymph nodes. Other organs, such as the liver and the spleen, are less commonly involved. Patients usually present with mild nonspecific symptoms. On imaging, hepatosplenomegaly with or without multiple focal lesions within the spleen may be seen in the active disease stage. Rarely, the disease may evolve to cirrhosis and liver failure. We report such a rare case of hepatosplenic sarcoidosis complicated by acute esophageal bleeding due to portal hypertension.

## Case report

A 60-year-old women was admitted with fatigue and abdominal discomfort. Further clinical history was unremarkable. Physical examination revealed epigastric tenderness. All laboratory test results were within normal range except for an elevated angiotensin-converting enzyme (ACE) 601 U/L (normal value: 115–419 U/L).

**Figure 1:**
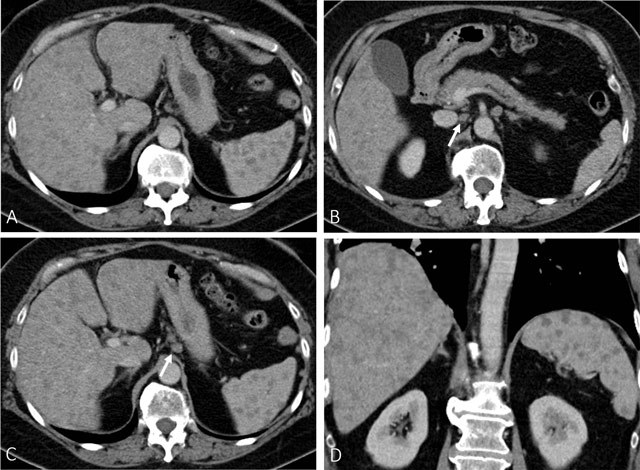
Axial (A, B, and C) and coronal (D) contrast-enhanced CT of the abdomen in the portal venous phase at time of the initial diagnosis. (A) shows multiple hypodense lesions throughout the liver and the spleen. (B) and (C) demonstrates lymph nodes of intermediate size (white arrow) at the retroperitoneum and along the lesser curvature of the stomach. The liver is slightly enlarged.

**Figure 2:**
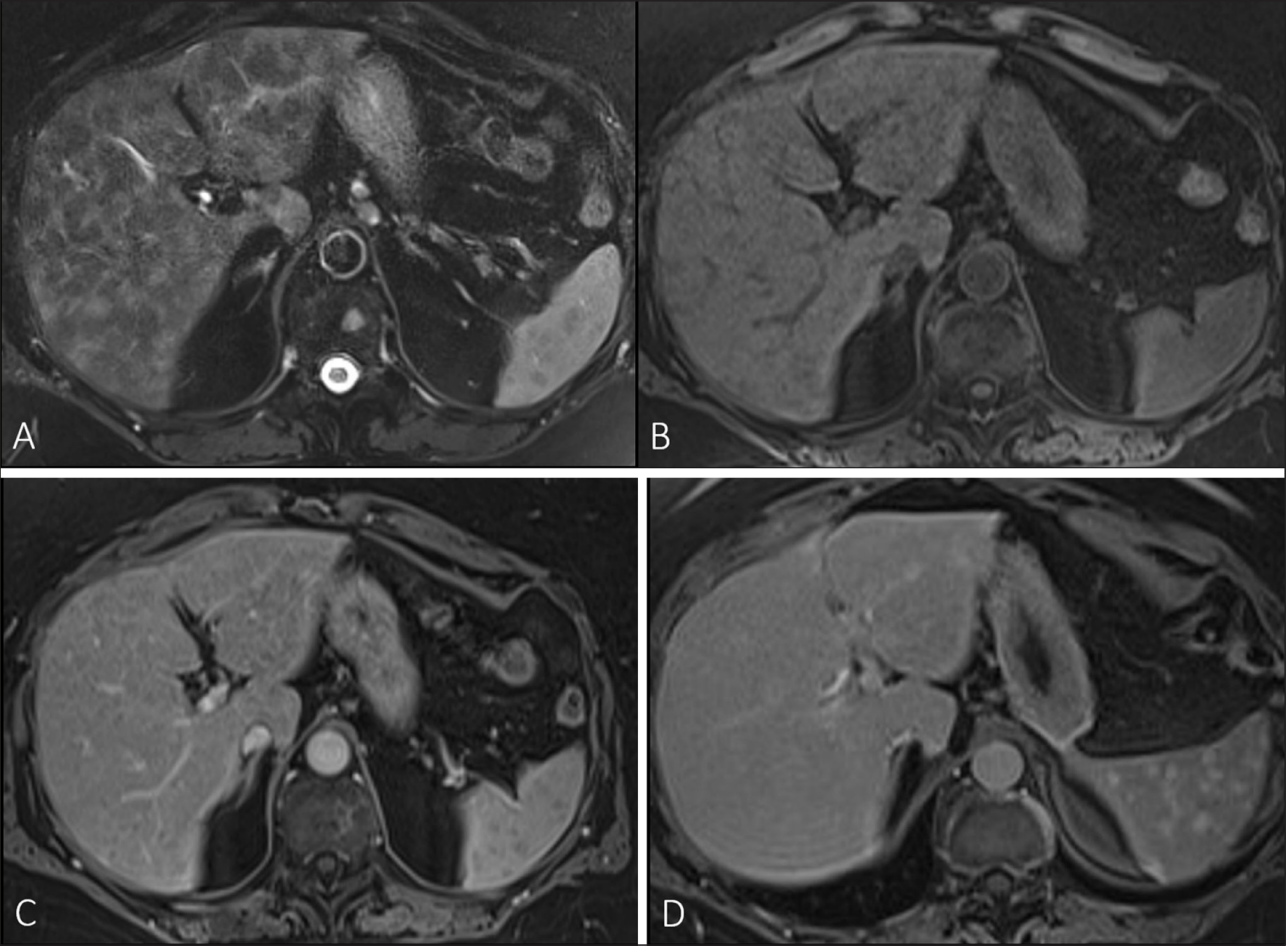
MRI of the upper abdomen at time of the initial diagnosis. Axial fat suppressed (FS) T2-weighted image (WI) (A), shows multiple hypointense liver and splenic lesions. Axial FS T1-WI before (B) and after administration of gadolinium-BOPTA contrast (C), show multiple hypointense focal lesions throughout the liver and spleen enhancing less than the surrounding liver and splenic parenchyma. FS T1-WI 1 hour after administration of gadolinium-BOPTA (D) shows delayed enhancement of the splenic lesions, whereas the liver lesions are not visible against the background of liverspecific enhancement of the normal liver.

Computed tomography (CT) (Fig. 1) and subsequent magnetic resonance imaging (MRI) (Fig. 2) were performed. Both techniques showed multiple focal lesions of intermediate size throughout the liver and spleen. Moderately enlarged lymph nodes were seen at the retroperitoneum and the lesser curvature of the stomach. Contrast-enhanced MRI after gadolinium-BOPTA administration showed hypo-enhancing lesions in the liver and spleen in the portal venous phase, whereas delayed phase one hour after injection could only demonstrate persistent enhancement of the splenic lesions. CT of the thorax was non contributive, as it revealed only slightly enlarged mediastinal lymph nodes and questionable thickening of the pleural fissures.

Although not supported by typical chest involvement, elevated ACE levels in combination with the imaging features of the upper abdominal organs were highly suggestive for hepatosplenic sarcoidosis. Unfortunately, the patient refused diagnostic liver biopsy at that time and was lost from follow-up.

**Figure 3:**
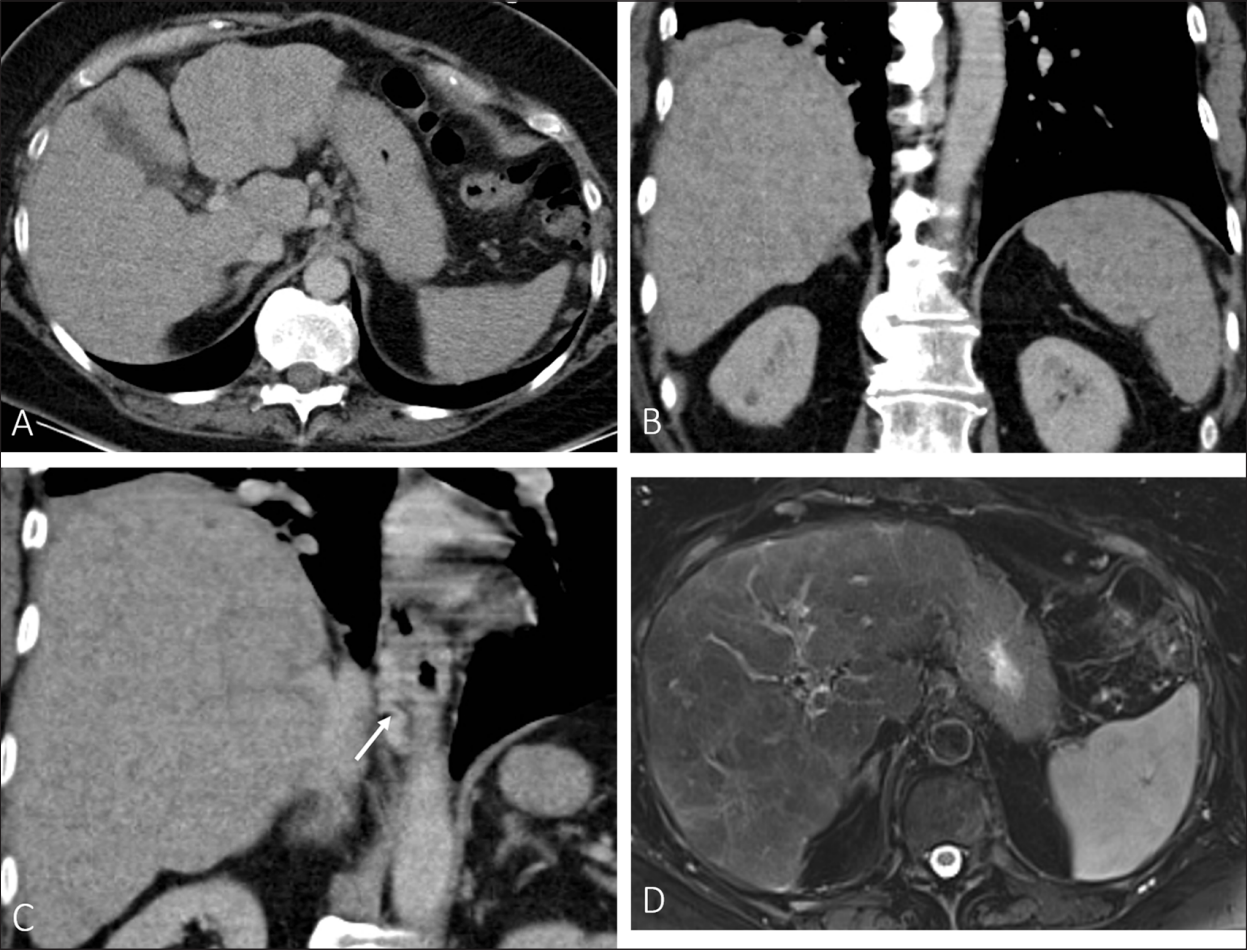
Imaging examinations four years later: Axial (A) and coronal (B and D) contrast-enhanced CT of the abdomen in the portal venous phase. (A) shows a nodular contour of the liver with a relative enlarged caudate lobe. (B) shows multifocal hypodense splenic lesions. (C) demonstrates esophageal varices (black arrow). Axial FS T2-WI (D) shows a heterogeneity of the liver parenchyma with multiple relative hypointense lesions diffuse throughout the liver.

**Figure 4:**
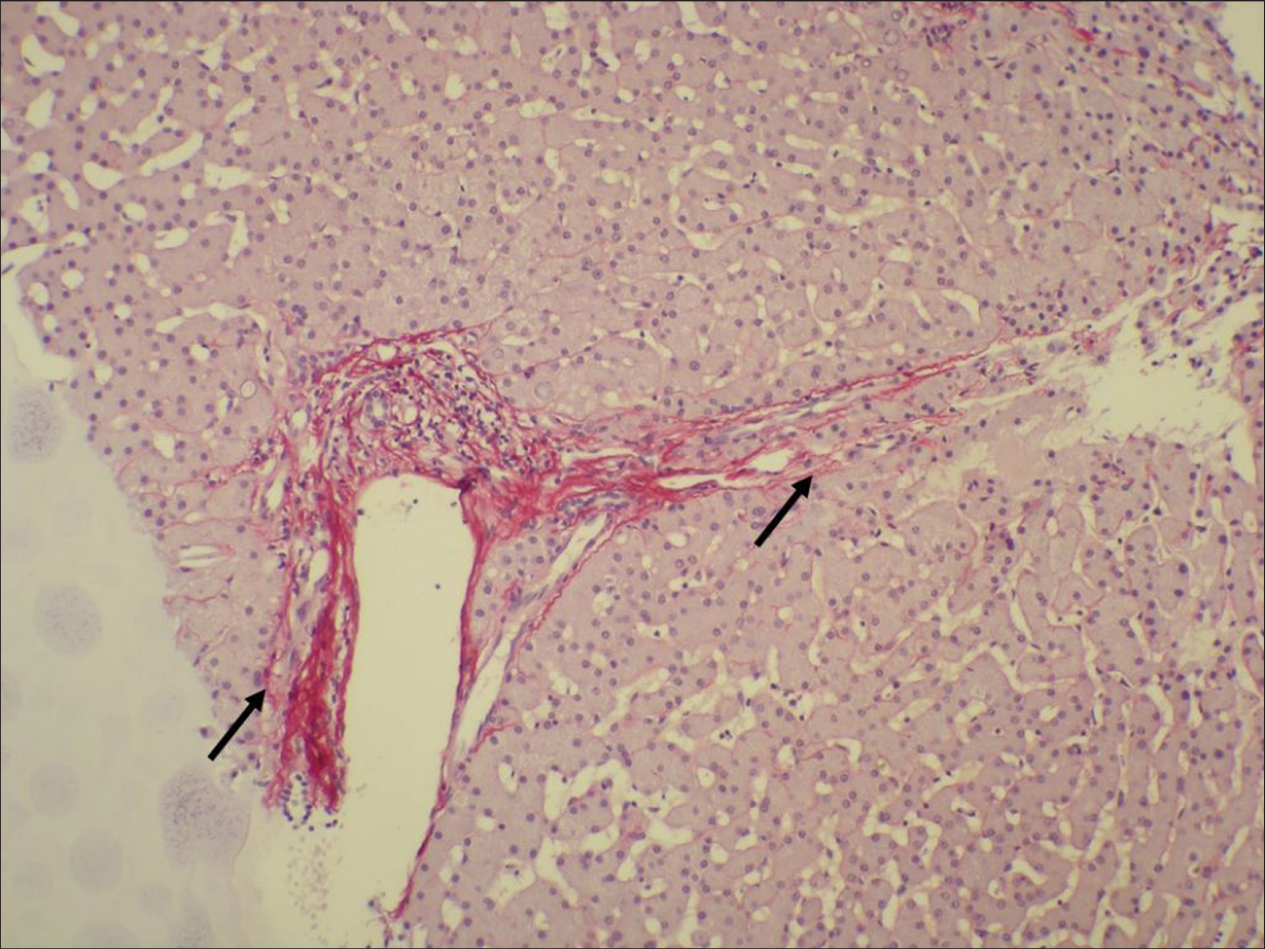
Sirius red staining. Portal tract is elongated due to connecting periportal fibrosis in keeping with stage F3 liver fibrosis (Metavir classification).

Four years later, she presented with esophageal variceal bleeding. The varices were ligated and the patient was stabilized. CT and MRI (Fig. 3) showed focal enlargement and irregular delineation of the caudate lobe and esophageal varices most compatible with liver cirrhosis. An ultrasound-guided biopsy showed periportal fibrosis (Fig. 4) in keeping with extinguished sarcoidosis. Because of the chronic nature of the disease, no further medical treatment with corticosteroids was initiated and waitful watching was recommended.

## Discussion

Sarcoidosis is a multisystemic disease characterized by formation of non-caseating granulomas in various organs. The etiology is still debated, although many authors suggest a combination of genetic susceptibility and environmental factors [1].

Sarcoidosis has a global prevalence of 1–40 per 100 000, with the highest prevalence’s observed in Scandinavian and African-Americans. It has a peak incidence between the third and fifth decade with a slight female predominance [1,2].

Lungs and mediastinal lymph nodes are involved in 90% of the cases. Although the liver is the third most affected organ, hepatosplenic sarcoidosis without lung involvement is uncommon [3].

The majority of the patients are asymptomatic or have mild, non-specific symptoms such as nausea, vomiting, weight loss and abdominal discomfort.

Laboratory findings are non-specific as well. Non-complicated patients with sarcoidosis have elevated ACE levels in 60% of cases. Thirty-five percent of patients have abnormal liver function tests, including raised alkaline phosphatase and gamma-glutamyltranspeptidase [4].

As the majority of patients with hepatosplenic sarcoidosis have chest involvement, plain radiography or even better CT of the chest are recommended as the next step in the diagnostic algorithm of suspected hepatosplenic sarcoidosis. Although hepatosplenic involvement is relatively frequent in patients with sarcoidosis, imaging of the liver and spleen is often normal. Only a minority of patients will present with abnormal findings on medical imaging. The most common imaging finding in hepatosplenic sarcoidosis on ultrasound consists of hepatomegaly with or without retroperitoneal adenopathy. CT and T2-weighted MRI are more sensitive to detect non-caseating granulomas as multiple hypodense or T2-hypointense nodules of intermediate size in 5–15% of cases [5]. These nodules show delayed contrast enhancement in the portal venous phase on both CT and MRI.

An additional point of interest in our case is the enhancement pattern of sarcoid lesions after Gd-BOPTA administration in the late phase imaging. Gd-BOPTA is an MR contrast agent with partial nonspecific and liverspecific effect. Delayed enhancement of the sarcoid lesions compared to the normal splenic parenchyma results in a high contrast between the lesions and the normal spleen. On the contrary, accumulation of Gd-BOPTA in the hepatocytes and bile ducts of the normal liver parenchyma hampers lesion conspicuity of hepatic sarcoid lesions in the hepatospecific phase.

The differential diagnosis of multifocal hepatic and splenic lesions includes metastases, multifocal lymphoma and fungal infection. Metastases should be considered as the preferred diagnosis if the patient has a known malignancy. In multifocal liver lymphoma, focal hepatosplenic lesions are usually larger than in sarcoidosis and are associated with more bulky adenopathy. Hepatosplenic lesions in fungal infection are typically smaller than in sarcoidosis and patients are often immunocompromised and are critically ill [6].

In only 1% of cases hepatosplenic sarcoidosis may be complicated by portal hypertension, cirrhosis and liver failure due to periportal fibrosis [7]. Histopathology is required for definite diagnosis of hepatosplenic sarcoidosis.

Medical treatment of active hepatosplenic sarcoidosis consists of prednisone 20–40 mg/day. Since most patients are asymptomatic or have only mild symptoms, potential side effects and therapeutic benefits of corticosteroids should be outweighted carefully [8,9].

## Conclusion

Although sarcoidosis of the liver and spleen is well-known disease, symptomatic hepatosplenic sarcoidosis evolving to end stage liver disease is extremely rare and therefore a challenging diagnosis. The radiologist should consider this rare diagnosis in the appropriate clinical setting.

## Competing Interests

[[COMPETING INTEREST STATEMENT TO BE PROVIDED]]
